# Speech recognition according to the length of hearing aid use

**DOI:** 10.1590/S1808-86942010000400010

**Published:** 2015-10-19

**Authors:** Tiago Petry, Sinéia Neujahr dos Santos, Maristela Julio Costa

**Affiliations:** a Master's degree in human communicaton disorders, Santa Maria Federal University. Assistant professor of the speech therapy course at the Feevale University Center; b Master's degree in human communicaton disorders, Santa Maria Federal University. Clinical speech therapist; c Doctoral degree in human communicaton disorders, Sao Paulo Federal University. Adjunct professor of the speech therapy department, Santa Maria Federal University. Santa Maria Federal University

**Keywords:** audiometry, hearing aids, speech

## Abstract

The use of hearing aids can provide plasticity to the hearing system as well as improve speech recognition as time goes by.

**Aim:**

To compare the influence of the length of hearing aid use on the benefit obtained with the hearing aids in adults and the elderly, new hearing aids users.

**Materials and methods:**

Prospective study with 40 individuals with mild to moderate-severe sensorineural hearing loss, gathered in 2 groups: Adults Group – 13 people aged between 28 and 59 years old; and Elderly Group – 27 people aged between 61 and 78 years old. These people were assessed 14 and 90 days after hearing aid fitting. Sentence recognition threshold in silence and under noise as well as the percentage indexes of sentences recognition in silence and under noise were obtained.

**Results:**

The comparison between values obtained after 14 and 90 days of hearing aid use did not show statistically significant differences. When comparing values between the groups, no statistically significant difference was observed either.

**Conclusion:**

We did not find influences of the length of hearing aid use and the benefit obtained from using them; the results achieved by adults and the elderly were similar.

## INTRODUCTION

Peripheral hearing loss affects hearing as a whole. Not only is there a quantitative decrease in sound, but also loss of speech recognition, which causes a negative social and emotional impact that affects the quality of life.

Adaptation of hearing aids is the therapy of choice when hearing loss can no longer be corrected by medication or surgery. Hearing aids are important not only for communication and spatial orientation but also because hearing reaffirms an individual's existence as a human being.^1^

Hearing aids raise the intensity of ambient sounds, thereby increasing auditory stimulation. Such stimulation may foster plasticity in the auditory system and in time improve speech recognition.^2^

The time period related to improved performance in speech recognition tests for adaptation of a hearing aid, as subjects learn to use new available speech cues with amplification, is named perceptual acclimatization.^3^

The effect of perceptual acclimatization is to systematically change hearing performance with time; this is unrelated to changes in available acoustic information for hearing aid users.^4^

In hearing aid performance tests, using words and sentences is an approximate representation of daily speech conditions. One of the most important features to be measured in human hearing is the ability to understand speech, which makes it possible to assess receptive communicative function and yields data about how a subject operates in daily hearing situations.^5^

The Portuguese Sentence List (PSL) test^6^ uses sentences as a stimulus and assesses speech recognition in silence and in the presences of competitive noise; it may be applied in a clinical setting or for several purposes in research.

A period of use of hearing aids is needed to reestablish speech abilities and for the benefits of hearing aids to be evaluated; it is also essential to carry out studies aiming at verifying the effects of perceptual acclimatization to monitor the development of hearing in a new hearing aid user.^7^

Based on this background, the purpose of this study was to assess young and elderly subjects with mild to moderately severe sensorineural hearing loss, new to hearing aids, by applying the PSL test,^6^ to verify the effect of time of use of amplification on the benefit gained from using hearing aids; and secondly, to investigate whether there were differences between adult and elderly patients in our results.

## MATERIAL AND METHOD

This study was carried out at the Hearing Aid Laboratory (Laboratório de Próteses Auditivas) of the institution of origin. It was registered at the Project Office under the number 019731 and approved by the institutional review board (certificate number 0138.0.243.000-06). All participants signed a free informed consent form after receiving information about the purpose of the study and its method. The inclusion criteria were:

• Age equal to or over 18 years;
• An audiological diagnosis of mild to moderately severe sensorineural hearing loss,^8^ with an onset in the post-lingual period;
• A speech recognition threshold equal to or below 65 dB SL in the best ear.
• Having been referred for use of binaural hearing aids;
• Not having started using hearing aids;
• No factor that might interfere with the test, such as neurological conditions and/or altered verbal fluency;

From January to October 2008, subjects that visited the Hearing Aid Laboratory and met the inclusion criteria were preselected to start hearing aid selection and adaptation procedures. Of 210 patients, 47 were preselected. Among these patients, those with other health conditions or any other impediment to returning for a second evaluation were excluded. Thus, of 47 preselected subjects, 40 were fully evaluated and comprised the sample.

The 40 subjects were grouped according to age, as follows:

1 Group A (Adults [18 to 59 years]): 13 new users of hearing aids aged from 28 to 59 years (mean age = 48.77 years), of which four were male and nine were female.
2 Group I (Elderly [over 60 years]): 27 new users of hearing aids aged from 61 to 78 years (mean age = 68.85 years), of which 12 were male and 15 were female.

Assessments and reassessments were done from January 2008 to January 2009. All subjects undertook investigation of:

• SRTS - sentence recognition threshold in silence;
• SRTN - sentence recognition threshold in noise;
• SRPRS - sentence recognition percentage rate in silence;
• SRPRN - sentence recognition percentage rate in noise.

Free field tests were carried out in two evaluation sessions: 1st evaluation 14 days after hearing aid adaptation; 2nd evaluation 90 days after hearing aid adaptation.

The same evaluation period was applied for each patient.

Subjects used hearing aids with the setting applied by the technical team in charge of patients at the Hearing Aid Laboratory in both evaluation sessions; there were no changes between each.

A clinical history based on a closed question questionnaire was taken prior to the first evaluation for information about personal data, hearing complaints, the otological history, daily life habits, and education level. The results of pure tone audiometries, speech recognition threshold tests, and speech recognition percentage rates were recorded; these had served as proof of hearing loss and were the baseline for hearing aid programming.

SRTS, SRTN, SRPRS and SRPRN were tested with the PSL test,^6^ which consisted of a list of 25 sentences,^9^ seven lists with 10 sentences,^10^ and speech spectrum noise.^11^ Sentences and noise were on a CD in independent channels, and were presented using a CD player couples to an audiometer. Results were recorded in a standard protocol.

The test was applied in an acoustic booth in free field; subjects were facing the source of sound one meter away, at 0°° – 0°° azimuth. The application sequence in the 1st and 2nd evaluations was as follows:

• Presentation of sentences 1 to 10 of list 1A without competing noise to familiarize subjects with the test.
• Presentation of list 5B without competing noise to measure the SRTS.
• Presentation of list 6B without competing noise and with speech at 65 dB A to measure the SRPRS.
• Presentation of sentences 11 to 20 of list 1A with competing noise at 65 dB A to familiarize subjects with the test.
• Presentation of list 1B, with competing noise at 65 dB A to measure the SRTN.
• Presentation of list 2B, with fixed competing noise and speech at 65 dB A resulting in a S/N ratio equal to zero to measure the SRPRN.

The choice of lists was made because two tests with different aims were being conducted concomitantly. Selecting lists 1B, 2B, 5B and 6B resulted in no test list being repeated under similar conditions in any test.

The sentence presentation technique was based on a sequential, adaptive or ascending-descending strategy^12^ to measure the speech recognition threshold, which is the necessary level for a subject to correctly identify about 50% of presented speech stimuli.

The procedure for measuring thresholds consisted of presenting a stimulus under a certain condition with or without competing noise. If a subject was able to correctly recognize the speech stimulus, its intensity was decreased at preset intervals. Otherwise its intensity was increased. This procedure was repeated until the end of the list.

Based on the literature, 4 dB intervals were used until the first change in the type of response; additional stimulus presentation intervals differed by 2 dB until the end of the list.^12^

For the thresholds, the mean values were calculated from the presentation intensity of sentences at which the first response change occurred.

For percentage rates, the intensity was fixed throughout the sentence list; all correctly answered sentences were added up, which corresponded to 10 percentage points per sentence of the list.

Measurements were taken in free field after calibrating the equipment according to the characteristics of the test signal and the ambient acoustic conditions. The sound pressure level at which subjects perceived speech and noise were set during calibration. A digital Radio Shack sound pressure-measuring device was used for this purpose; it was placed one meter from the loudspeaker at a midpoint for both ears. An A6 measuring scale was used, which is adequate for measuring continuous noise and extreme intermittent noise values.

The sentence presentation intensity was gauged based on a recorded pure tone on the CD channel in which the sentences were recorded. This pure tone was a continuous reference tone, and was applied to maintain constant presentation conditions. This is because speech signals are complex sounds with a 30 dB variation between most and least intense sounds, oscillating 12 dB over and 18 dB below the mean;^13^ thus the need for a reference sound.

Before starting the evaluations, each CD output channels were gauged using the audiometer VU-meter. For this purpose a 1,000 Hz tone present in one channel, and a marking noise present in the other channel, were set at zero. Previous studies have noted that sentences had been recorded on the CD at a mean intensity of 7 dB below a pure tone intensity.^14^ This difference was taken into account and corrected on the device dial when applying the tests.

Measurements were taken in an acoustic booth; a Damplex DA65 model dual-channel digital audiometer and a TA 1010 model amplification system for free field audiometry were used. Sentences were presented using a Britania B5279 model CD player with the lineout option coupled to the audiometer.

Data were analyzed descriptively and statistically treated by considering the behavior of variables, comparing the results of evaluations at two different times for each group. Wilcoxon's test was applied, as it does not require data to have a normal distribution; it also tested whether the differences of two related values were statistically significant.

Differences between groups were verified for each evaluation. The Mann-Whitney test was applied for this purpose; it tested whether two independent variables not distributed normally had statistically significant differences. The significance level was 5% for both tests.

## RESULTS

Table 1, Table 2, Table 3, Table 4 show the results of the SRTS, SRTN, SRPRS and SRPRN tests, respectively, and the values of the Wilcoxon test at a 5% significance level, to compare the adaptation of hearing aids in adult and elderly subjects after 14 and 90 days.

**Table 1:** Descriptive measurements and the p-value of the SRTS on the 14th and 90th day after adaptation of hearing aids in groups A and I.

SRTS	n	Mean (dB A)	Lower Limit (dB A)	1^st^ Quartile (dB A)	Median (dB A)	3^rd^ Quartile (dB A)	Upper Limit (dB A)	p-value
Group A								
14 days	13	46,00	34,33	40,22	47,00	51,00	61,29	0,3821
90 days	13	46,00	32,20	39,80	44,50	53,33	60,78	
Group I								
14 days	27	46,58	35,20	40,78	44,50	51,28	76,67	0,9234
90 days	27	45,92	34,40	40,78	44,50	49,86	69,22

**Table 2:** Descriptive measurements and the p-value of the SRTN, at a 65 dB noise level, 14 and 90 days following adaptation of hearing aids for groups A and I

SRTN	n	Mean (dB A)	Lower Limit (dB A)	1^st^ Quartile (dB A)	Median (dB A)	3^rd^ Quartile (dB A)	Upper Limit (dB A)	p-value
Group A								
14 days	13	63,38	60,00	61,50	62,50	64,00	68,00	0,8887
90 days	13	63,39	56,71	61,44	63,22	64,11	70,56	
Group I								
14 days	27	64,36	58,50	62,50	64,11	66,33	76,67	0,9234
90 days	27	64,54	59,57	62,00	63,50	66,00	75,89

**Table 3:** Descriptive measurements and the p-value of the SRPRS, 14 and 90 days following adaptation of hearing aids for groups A and I

SRPRS	n	Mean (%)	Median (%)	p-value
Group A				
14 days	13	98,46	100	0,3173
90 days	13	100	100	
Group I				
14 days	27	94,44	100	0,5639
90 days	27	95,18	100

**Table 4:** Descriptive measurements and the p-value of the SRPRN, at a 65 dB noise level, 14 and 90 days following adaptation of hearing aids for groups A and I

SRPRN	n	Mean (dB A)	Lower Limit (dB A)	1st Quartile (dB A)	Median (dB A)	3rd Quartile (dB A)	Upper Limit (dB A)	p-value
Group A								
14 days	13	70,00	00	60	80	90	100	0,2457
90 days	13	75,38	40	60	80	90	100	
Group I								
14 days	27	70,74	10	50	80	90	100	0,6524
90 days	27	68,52	00	60	70	90	100	

Table 5 shows differences between values for adult and elderly subjects, for all the study variables. The Mann-Whitney test was used at a 5% significance level.

**Table 5:** P-value in a comparison of groups A and I for the SRTS, SRPRS, SRTN and SRPRN after 14 and 90 days following adaptation of hearing aids

Variables	Group A versus	Group I p-value
	SRTS	0,9080
14^th^ day	SRPRS	0,7185
	SRTN	0,2914
	SRPRN	0,9414
	SRTS	0,9309
90^th^ day	SRPRS	0,3203
	SRTN	0,3858
	SRPRN	0,4924

## DISCUSSION

Table 1 (SRTS) and Table 2 (SRTN) show that there were no statistically significant differences in thresholds after 14 and 90 days of adaptation in Group A and Group I. This demonstrates that at 14 to 90 days after hearing aid adaptation there was no speech recognition improvement in adult and elderly subjects.

According to the literature, speech recognition improvements in perceptual acclimatization are not evaluated immediately in new hearing aid users.^15^ Furthermore, the benefits of hearing aids are hugely affected by individual characteristics such as personality, motivation and expectations; they also depend on the acoustic ambience within which each subject is present.^16^

A study of the perceptual acclimatization phenomenon in adult new hearing aid users^2^ found that the mean speech recognition percentage rate improved after four and 16/18 weeks of amplification use, although this difference was not statistically significant. The authors concluded that the perceptual acclimatization phenomenon could not be assessed by using the speech recognition percentage rate. They also asked whether hearing aid users in fact perceived perceptual acclimatization and whether such individuals felt any benefit thereof. A longer follow-up period was recommended to assess the benefits by applying subjective measures and electrophysiological tests. It was also essential to consider auditory training as a directly supportive measure for improved speech recognition.^2^

Another study aimed to verify possible evidences of functional plasticity in the auditory system;^17^ its results were consistent with the effects of auditory perceptual acclimatization, thereby suggesting that hearing aid adaptation induces functional plasticity of the auditory system.^17^

The PSL^6^ test was applied in a study7 that monitored the first three months of new hearing aid users, and found perceptual acclimatization. The monosyllable speech recognition percentage rate and the SRTN with sentences and 65 dB fixed noise were investigated. This study revealed that speech recognition improved only 30 days after using sound amplification, and that the progression of speech abilities became optimal 60 days after adaptation. Thus, perceptual acclimatization only begins after the first month of adaptation, it is progressive, and results from using acoustic cues provided by hearing aid use.^7^

Our study generally showed no significant differences in the benefits gained with hearing aids; at an individual level, however, we found that 53.85% of adults and 48.15% of elderly patients had some sign of improvement in SRTS and SRTN when comparing the evaluation and reevaluation tests. Among adult subjects that improved, mean changes were 2.80 dB for the SRTS and 1.19 dB for the SRTN. In elderly subjects, mean improvements were 4.29 dB (SRTS) and 1.86 dB (SRTN). With such differences, we agreed with the influence of individual and environmental peculiarities,16 and that such factors partly explain why amplification induced functional plasticity in some of our subjects, albeit poorly perceived, but not in others. Some of our subjects mentioned that they were using hearing aids continuously and were pleased with its benefits; they may have done so because the whole process involves undertaking specialized tests, hearing aid selection adaptation, and clinical visits free of charge. Thus, our patients may have involuntarily taken on a satisfied attitude when in reality they may not have assumed their right to make complaints. Statements along these lines in subjective assessments have been made, where patients reveal attitudes of humbleness and gratitude for having been given hearing aids free of charge, and do not feel it right to complain.^7^

Auditory perceptual acclimatization is a systematic change in the auditory system with time.^18^ There is an evident conflict about the existence of perceptual acclimatization,^19^ as some studies have shown improved performance while other have failed to demonstrate the effects of auditory perceptual acclimatization. There are at least three explanations for the studies that did not find perceptual acclimatization.^19^ Firstly, subjects may have had few opportunities for improvement because of minor hearing losses,^20^ or may have had prior experience with hearing aids,^21^ or may have used hearing aids in a limited manner.^20^^,^^21^ Secondly, negative findings may occur if the methods fail to measure the changes that actually occur.^22^ Thirdly, findings may attempt to demonstrate perceptual acclimatization based on inappropriate levels in test materials, and thereby often fail.^19^

Based on these statements, it is possible to question the effective use of hearing aids during our study period in adult and elderly subjects that showed no change in speech recognition with time. If about 50% of subjects showed improved SRTS and SRTN tests, then why did the other half show not changes or even worse results? Again we respect individualities, but raised the hypothesis that most of the patients in this study used hearing aids only during short periods during the first months after adaptation, even after receiving proper guidance and having free access to speech therapists for consultation. We also conjectured whether a hearing training program for hearing aids to specifically encourage speech recognition and intelligibility abilities would have yielded superior results.

We emphasize that the sensitivity of the PSL test6 (used in this study) for proving the effects of perceptual acclimatization has been demonstrated in another study; we believe that the method for measuring the SRTS and/ or SRTN was not a determining factor to not generally demonstrate the effects of perceptual acclimatization.

Analysis of the SRPRS (Table 3) and the SRPRN (Table 4) demonstrated no statistically significant differences in comparison results between the 14th and 90th days after adaptation in both Group A and Group I.

We chose to fix the intensity of speech at 65 dB for measuring the SRPRS. As such, the method allowed most subjects to attain maximum scores at the first evaluation, thereby not permitting any to reach superior scores in the reevaluation. We felt that this procedure was not valid for verifying the influence of time of amplification use on the benefits gained from hearing aids. We therefore suggest that the SRTS be used as a basis for choosing at which level the intensity should be fixed for measuring the SRPRS.

Concerning the SRPRN, subjects were evaluated under fixed noise and speech stimuli at 65 dB A, a S/N ratio equal to zero. Our analysis showed that no improvements in speech recognition occurred in adult or elderly subjects from days 14 to 90 after amplification use.

As with SRTS and SRTN results, SRPRN values differences were not statistically significant in our overall evaluation. At an individual level, however, 46.16% of adults and 37/04% of elderly subjects had, respectively, intermediate changes of 20% and 21% in their SRPRN results. The same comments made above apply here: individuality, environmental influences, and limited use of hearing aids.

A study that aimed to show SRPRN changes as the S/N ratio was altered^23^ showed that a 1.0 dB variation in the S/N ratio, in free field, represented a 12.12% change in the SRPRN of normal-hearing subjects, while this same variation in the S/N ratio caused an 11.20% change in the SRPRN of hearing loss subjects. If in our study some adult and elderly subjects had mean respective improvements of 1.19 and 1.86 dB in their SRTN values, their improvements in SRPRN values may be thus explained. We raised the possibility of clinically important alterations in these subjects.

We found no papers in the literature associating sentence recognition percentage rates gathered by PSL testing with the effects of perceptual acclimatization.

We also investigated any differences between Group A and Group I results for all evaluations and reevaluations. Table 5 shows that no group was superior to the other in any evaluation.

Although subjects in both groups had similar hearing losses in degree and configuration, we expected Group I to yield worse results than Group A, especially because of the aging process. Our results, however, indicated that adult and elderly subjects performed similarly.

Although this study did not yield statistically significant results, the effects of hearing performance with time should never be underrated when adapting hearing aids. We also emphasize the importance of hearing training, regardless of age, as an ally for improved speech recognition.

## CONCLUSION

A critical analysis of results showed that:

• No time of use of amplification effect on benefits of hearing aids was found in adult and elderly subjects, based on the PSL test.
• Adult and elderly subjects had similar SRTS, SRTN, SRPRS and SRPRN results, after 14 and 90 days of hearing aid use.
